# Magnetic resonance imaging R2* sequences can better detect microstructural cartilage changes than T2 mapping in *cynomolgus* monkeys with limited knee kinematics: preliminary imaging findings

**DOI:** 10.1186/s12891-022-05817-5

**Published:** 2022-09-17

**Authors:** ManMan Gao, JianMin Wang, LuoYong Jiang, XiMin Pan, Federico Canavese, YiQiang Li, WenTao Wang, ZhiYu Zhou, WeiMin Zhu

**Affiliations:** 1 Department of Sport Medicine, Inst Translat Med, The First Affiliated Hospital of Shenzhen University, Shenzhen Second People’s Hospital, 3002nd SunGangXi Road of FuTian District, Shenzhen, 518025 China; 2 Innovation Platform of Regeneration and Repair of Spinal Cord and Nerve Injury, Department of Orthopaedic Surgery, The Seventh Affiliated Hospital, Sun Yat-Sen University, 628th ZhenYuan Road of GuangMing District, Shenzhen, 518107 China; 3 Guangdong Provincial Key Laboratory of Orthopedics and Traumatology, The First Affiliated Hospital of Sun Yat-Sen University, Guangzhou, 510080 China; 4 Shenzhen Key Laboratory of Anti-Aging and Regenerative Medicine, Department of Medical Cell Biology and Genetics, Health Sciences Center, Shenzhen University, Shenzhen, 518061 China; 5 Department of Orthopedics, Inst Translat Med, The First Affiliated Hospital of Shenzhen University, Shenzhen Second People’s Hospital, Shenzhen, 518025 China; 6 Department of Radiology, The Sixth Affiliated Hospital (Gastrointestinal Hospital), Sun Yat-Sen University, Guangzhou, 510655 China; 7 Department of Pediatric Orthopaedics, Lille University Center, Jeanne de Flandre Hospital, Avenue Eugène Avinée, 59037 Lille cedex, France; 8 Department of Pediatric Orthopaedics, GuangZhou Women and Children’s Medical Center, Guangzhou Medical University, Guangzhou, 510623 China

**Keywords:** Cartilage, R2*, T2 mapping, Range of motion, Microstructural change

## Abstract

**Background:**

The difference between MRI (Magnetic resonance imaging)-R2* and T2 mapping sequences regarding their superiority in the detection of microstructural cartilage changes in knees with limited ROM (range of motion) was unknown.

**Methods:**

Twenty male *cynomolgus* monkeys (mean age: 10.65 ± 0.97 years) underwent knee ROM evaluations and were divided into three groups: Group A (*n* = 10), with similar left and right knee ROM; Group B (*n* = 5), with left knee ROM superior to right; and Group C (*n* = 5), with left knee ROM inferior to right. Twenty-eight ROIs (regions of interest) in the cartilage of the lateral (L) and medial (M) femoral trochlea (FT), anterior (A)/central (C)/posterior (P) femoral condyle (FC) and tibial plateau (TP) of both knees were identified in each monkey. The corresponding ROI values in R2* and T2 mapping sequences were recorded for analysis. One-way ANOVA, Chi-square tests and Pearson’s correlation analysis were used for statistical analyses.

**Results:**

Among the total 1120 ROIs, significant differences in R2* values among the three groups existed in two ROIs: cartilage of the right MPTP (*F* = 5.216, *P* = 0.017) and left MAFC (*F* = 4.919, *P* = 0.021). However, the T2 mapping values of all ROIs were similar among the three groups. Microstructural cartilage changes occurred more frequently in the medial (40 ROIs) than in the lateral (0 ROIs) knee compartment (χ^2^ = 43.077, *P* < 0.001). The Group B cartilage R2* value of the right MPTP increased with the difference in bilateral knee ROM (*r* = 0.913, *P* = 0.030).

**Conclusions:**

In knees with limited ROM, MRI-R2* sequence is superior to T2 mapping in the detection of microstructural cartilage changes, which the medial knee compartment was more susceptible to. Cartilage R2* values tend to increase with the amount of knee ROM loss.

## Background

Altered knee kinematics and decreased joint range of motion (ROM) are usually caused by several factors such as microstructural changes in cartilage, synovial inflammation, and ligament stiffness [1, 2]. Microstructural changes in articular cartilage, one of the most common causes of limited ROM, tend to worsen in the absence of early diagnosis or appropriate intervention due to the inability of cartilage regeneration [1–3]. Magnetic resonance imaging (MRI), including T2 mapping and R2* sequences, is a common tool used to diagnose microstructural cartilage changes [4, 5]. However, which sequence shows better detective superiority in knees with limited ROM remains controversial.

T2 maps are quantitative sequences that are sensitive to the water content and collagen composition of cartilage [6, 7]. At present, most studies have identified the significance of T2 mapping in the detection of cartilage changes by intraoperative validation [8, 9]. However, these reports did not provide any information on the correlation between T2 mapping and the detection of microstructural cartilage changes in knees with limited ROM, which may progress without early diagnose [3, 8, 9].

Previous studies have reported the utilization of MRI-R2* sequences in the diagnosis of several disorders charactrerized by abnormal iron deposition, such as liver and brain lesions [10, 11]. Nevertheless, to our knowledge, no reports have investigated the detection of microstructural cartilage changes in knees with limited ROM using R2* sequences. This may be related to insufficient knowledge of the potential correlation between abnormal iron metabolism in chondrocytes and the progression of microstructural cartilage changes [12, 13]. Recently, Yao et al. [14] demonstrated that Recently, Yao et al. [14] demonstrated that chondrocyte ferroptosis and abnormal iron deposition could contribute to the development of microstructural changes in cartilage tissue, although no study has analyzed the MRI characteristics of this process in knees with limited ROM by R2* sequencing. Furthermore, the difference in the detection of microstructural cartilage changes between R2* and T2 mapping sequences in knees with limited ROM remains to be elucidated.

In this study, we aimed to evaluate cartilage measurements in T2 mapping and R2* sequences in *cynomolgus* monkeys with normal and reduced knee ROM and to compare T2 mapping with R2* sequences regarding their difference in the detection of microstructural cartilage changes associated with limited knee ROM. Our hypothesis is that R2* sequences can detect microstructural cartilage changes in knees with altered kinematics (decreased flexion–extension) and that cartilage R2* values increase in proportion to the amount of knee ROM loss.

## Methods

After securing the approval (no. GZZ20210126; Animal Experimental Ethical Inspection Form of Institute of Zoology, Guangdong Academy of Sciences), we randomly selected 20 male *cynomolgus* monkeys with an average age of 10.65 ± 0.97 years (range, 6 to 20), with a conversion ratio of 1:3.5 to equivalent human age (range, 21 to 70) [15, 16], and a mean weight of 8.03 ± 0.44 kg (range, 5 to 12).

*Cynomolgus* monkeys have been used as models for limited ROM in humans due to their human-like anatomy and walking ability [17]. Before the experiment started, the 20 monkeys were housed for 3 months in separate cages in an Association for Assessment and Accreditation of Laboratory Animal Care (AAALAC) International approved facility. Specifically, the monkeys were housed in a climate-controlled environment (21 °C temperature, 40%–50% relative humidity, 10–15 air changes per hour, and 12 h/12 h light/dark cycles) in stainless-steel cages. The cages measured 0.9 m (m) in length, 0.8 m in height, and 0.85 m in depth, and the cage volume was 0.612 m^3^. The monkeys were fed a standard pellet diet (Lab Monkey Diet; Fuda Biotechnology) and water ad libitum. A tattoo on the left ear served to identify the monkeys.

### Assessment of knee ROM

**Fig. 1:**
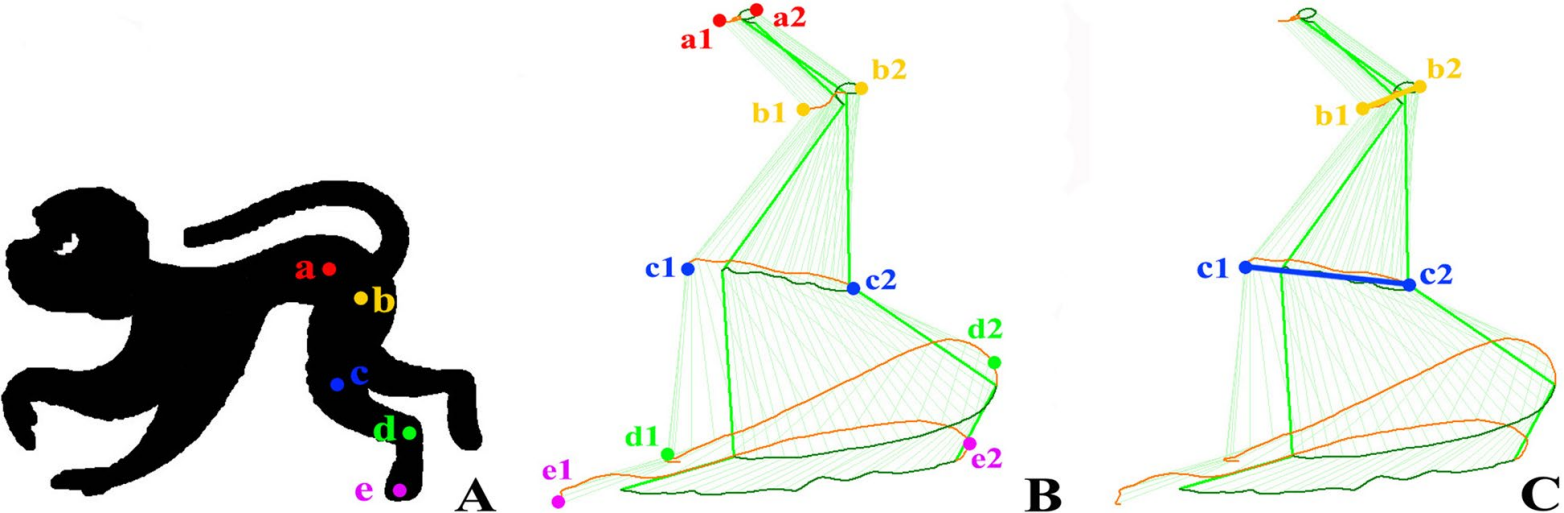
Hip and knee flexion–extension measurements in the sagittal plane during a walking trial. **A** five colored markers (red, yellow, blue, green, purple; a, b, c, d, e) were used to mark the sacroiliac, hip, knee, ankle, and toe joints, respectively; **B** the foremost (a1, b1, c1, d1, e1) and backmost (a2, b2, c2, d2, e2) positions the corresponding joints reached during walking; **C** the length of the yellow line b1-b2 and blue line c1-c2 were recorded as the range of motion of the hip and knee joints, respectively

All monkeys underwent knee ROM assessment using a three-dimensional GaitScan pedobarography system (Kinema Tracer, Kissei Comtec). The system consisted of two devices, including a treadmill (1540 mm × 690 mm) and a camera that captured kinematic data. One week (10 min per day) of walking training on the treadmill at a speed of 3 km per hour (3 km/h) was performed before knee ROM assessment. Five markers of different colors (red, yellow, blue, green and purple) were placed at the level of the sacroiliac, hip, knee, ankle and toe joints (Fig. 1). The camera could capture the kinematic data of different joints according to the amount of motion of the corresponding colored markers.

For the knee ROM assessment, the monkeys walked on the treadmill at a speed of 3 km/h. The camera captured the movements of the five colored markers at 30 frames per second; the data were then processed by the system and displayed as sagittal plane motion during a walking trial, as shown in Fig. 1. Kinematic parameters, including the duration of the stance and swing phases, step length, stride length, cadence, and step interval, were recorded. Six consecutive walking trials were captured for each monkey.

The ROMs of the hip and knee joints were recorded as the flexion–extension (FL-EX) movement distances, which were obtained from sagittal plane view of the walking trial (Fig. 1). The length between the foremost and backmost positions each joint achieved during a walking trial was recorded as the FL-EX movement distance (Fig. 1), which was expressed in centimeters (cm). The mean and standard error of the distance during six consecutive trials were used for the analysis.

**Fig. 2:**
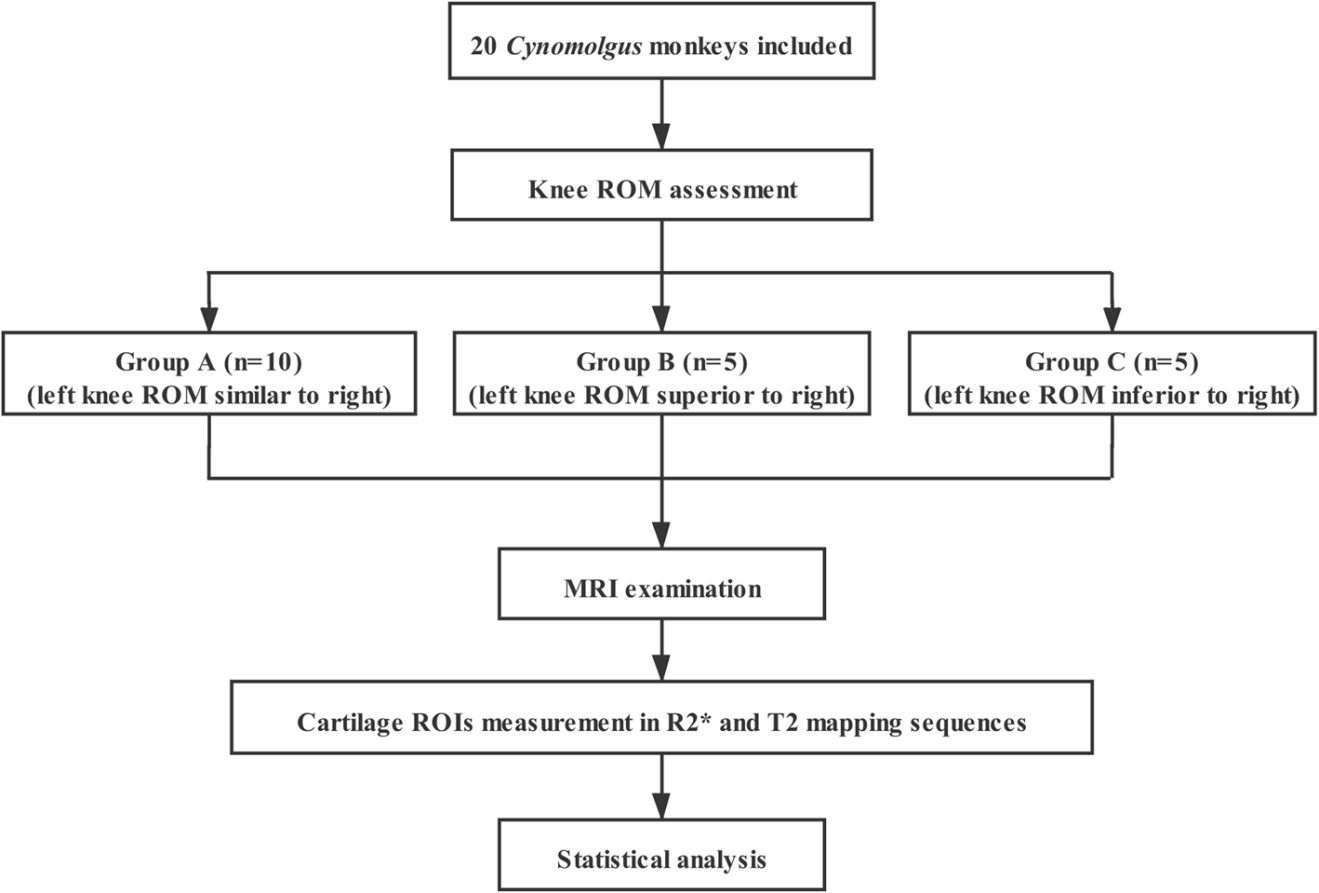
Study flowchart

According to the knee ROM, the 20 monkeys were divided into three groups: 1) Group A: left knee ROM similar to right knee ROM, with less than 1 cm difference in knee FL-EX between the two knees; 2) Group B: left knee ROM superior to right knee ROM (> 1 cm difference in knee FL-EX between the two knees); 3) Group C: left knee ROM inferior to right knee ROM (> 1 cm difference in knee FL-EX between the two knees) (Fig. 2).

### MRI examination

Magnetic resonance data were acquired using a 3.0-T MRI scanner (Discovery MR750 W, GE Healthcare).

Under general anesthesia (Zoletil 50, 10 mg/kilogram), images were acquired while the monkeys were in a lateral decubitus position with the knee joint placed in the center of the knee array coil. All sequences were acquired in the sagittal plane. T2 mapping images were acquired using a turbo spin echo sequence, while R2* images were obtained using an iterative decomposition of water and fat with echo asymmetrical and least squares estimation sequence. Table 1 shows the MRI parameters in detail (Table 1).

**Table 1:** MRI protocols

Sequence	R2*	T2 mapping
Pulse sequence	IDEAL-IQ	TSE
Repetition time (ms)	12.5	1200
Echo time (ms)	3.7	7.1, 14.1, 21.2, 28.2, 35.3, 42.4, 49.4, 56.5
Field of view (mm)	28 × 28	14 × 14
Pixel bandwidth (hz)	1388.9	651
Voxel size (mm)	1.8 × 1.8	0.7 × 0.7
Slice thickness (mm)	3.3	3
Interslice gap (mm)	0	0.3
Number of slices	8	14
Acquisition time (min)	00:26	08:24

*IDEAL-IQ* Iterative decomposition of water and fat with echo asymmetrical and least-squares estimation quantitation, *TSE* Turbo spin echo, *ms* millisecond, *mm* millimeter, *hz* Hertz

**Fig. 3:**
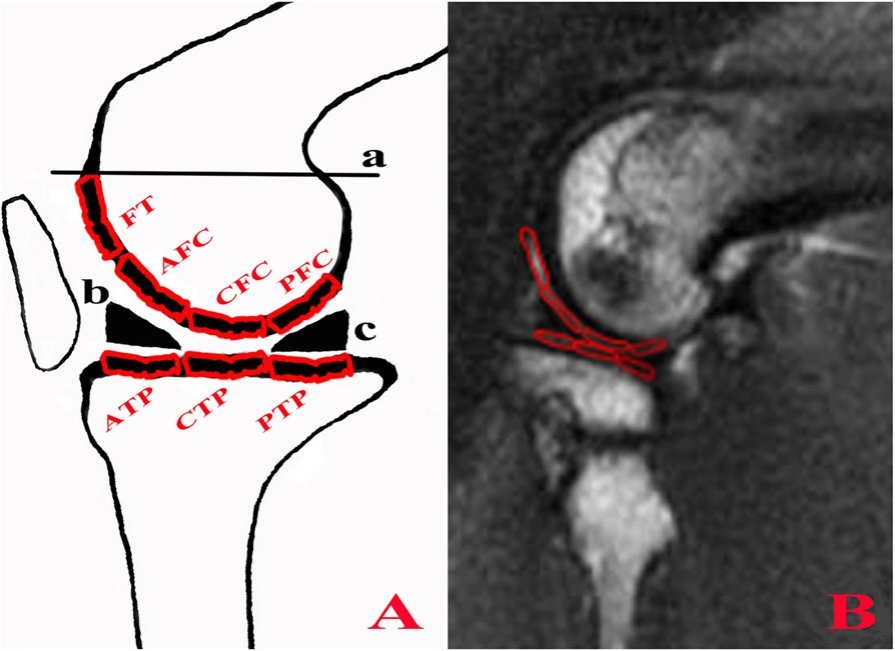
The definitions of cartilage ROIs (medial and lateral FT, AFC, CFC, PFC, ATP, CTP, PTP). **A** Line a was parallel to the tibial plateau (TP) articular surface and went through the posterior (P) femoral condyle (FC). The proximal part of the femoral trochlea (FT) was bordered by line a, and the distal part was bordered by the anterior (A) rim of the anterior menisci horn (triangle b). AFC and ATP were above and below triangle b, respectively. CFC and CTP were above and below the central (C) portion of the meniscus body, respectively. PFC and PTP were above and below the posterior menisci horn (triangle c), respectively. **B** The corresponding cartilage ROIs (FT, AFC, CFC, PFC, ATP, CTP, PTP) were marked on a morphological T2 sequence before copying to R2* and T2 mapping sequences

The MRI sequence data were then transferred to a workstation (AW4.7 version, GE Healthcare). For image acquisition, a femoral intercondylar notch was used as an anatomical landmark to differentiate the lateral and medial planes. Using the menisci as an anatomical reference, the anterior and posterior cartilage regions of interest (ROIs) of the femoral condyle (FC) and tibial plateau (TP) were defined as above and below the anterior and posterior menisci horn, respectively, and the central cartilage ROIs of the FC and TP were set above and below the menisci body (Fig. 3). The cartilage ROIs of the femoral trochlea (FT) were defined by the proximal part bordered by the line parallel to the TP articular surface and through the posterior FC and the distal part bordered by the anterior rim of the anterior menisci horn (Fig. 3).

A total of 28 cartilage ROIs were identified in both knees, including the right and left cartilage ROIs of the FT and the anterior (A)/central (C)/posterior (P) FC and TP in the lateral (L) plane (LFT, LAFC, LCFC, LPFC, LATP, LCTP, LPTP) and medial (M) plane (MFT, MAFC, MCFC, MPFC, MATP, MCTP, MPTP) (Fig. 3).

For the acquisition of ROI values, first, each cartilage ROI was marked on a morphological image of the T2 sequence, which provided the best cartilage-to-bone and cartilage-to-joint fluid contrast (Fig. 3). Subsequently, the ROIs were copied to T2 maps and R2* sequences and correctly positioned. Finally, the mean values of each cartilage ROI were recorded for both T2 mapping and R2* sequences. T2 mapping values were expressed in milliseconds, while R2* values were expressed in Hertz. MRI measurements were performed three times for each ROI, with a minimum interval of one week between measurements, and the mean and standard error were used for the final analysis.

### Statistical analysis

Statistical analysis was performed using the statistical package SPSS 13.0 (SPSS software, IBM).

Data are expressed as numerical variables, frequencies, and percentages, with means and standard errors. Paired-samples t tests were used to evaluate the difference between the right and left extremities regarding kinematic parameters, knee and hip ROM, and differences in acquisition time between T2 mapping and R2* sequences. One-way ANOVA was used to assess the differences among the three groups regarding age, weight, bilateral kinematic parameters, and the 28 cartilage ROIs values drawn from T2 mapping or R2* sequences. Chi-square tests were used to evaluate the differences regarding the number of positive ROIs reflected by T2 mapping or R2* sequences and the locations of microstructural cartilage changes in the medial and lateral knee compartments and in the right and left knees. Pearson’s correlation analysis was used to evaluate the correlation between right and left knee and hip ROM in each group and the correlation between the absolute difference in bilateral knee ROM and cartilage R2* values. Agreement as indicated by the intraclass correlation coefficient (ICC) was defined as follows: 0 to 0.4, fair agreement; 0.41 to 0.60, moderate agreement; 0.61 to 0.80, substantial agreement; and 0.81 to 1.00, excellent agreement. The level of statistical significance was set at *P* < 0.05.

## Results

### Assessment of knee ROM

A total of 20 *cynomolgus* monkeys underwent knee ROM evaluations. The kinematic parameters, including the stance phase duration (expressed as a percentage), swing phase duration (expressed as a percentage), step length, stride length, cadence and step interval, were similar between the right and left sides (*P* > 0.05) (Table 2). According to knee ROM, the twenty *cynomolgus* monkeys were divided into three groups: Group A (50%, *n* = 10), with an average absolute difference in FL-EX between the two knees of 0.4 ± 0.09 cm; Group B (25%, *n* = 5), with an average absolute difference in FL-EX between the two knees of 2.36 ± 0.32 cm (Left > Right); and Group C (25%, *n* = 5), with an average absolute difference in FL-EX between the two knees of 2.21 ± 0.09 cm (Left < Right). No significant differences existed among the three groups regarding age, weight or bilateral kinematic parameters (*P* > 0.05) (Table 2).

**Table 2:** Analysis of age, weight, and the kinematic parameters of both knees in three Groups

	Group A (*n* = 10)	Group B (*n* = 5)	Group C (*n* = 5)	F	*P*
Age (y)	9.30 ± 1.05	12.40 ± 2.66	11.60 ± 1.94	1.022	0.381
Weight (kg)	7.65 ± 0.54	8.00 ± 1.17	8.82 ± 0.87	0.564	0.579
Duration of stance (%)	-	-	-	-	-
Right	53.08 ± 0.92	50.87 ± 1.61	55.91 ± 3.64	1.384	0.278
Left	53.51 ± 1.09	55.02 ± 1.56	53.77 ± 1.90	0.290	0.752
t	0.285	2.693	1.173	-	-
P	0.782	0.054	0.306	-	-
Duration of swing (%)	-	-	-	-	-
Right	46.92 ± 0.92	49.13 ± 1.61	44.09 ± 3.64	1.384	0.278
Left	46.49 ± 1.09	44.98 ± 1.56	46.23 ± 1.90	0.290	0.752
t	0.285	2.693	1.173	-	-
P	0.782	0.054	0.306	-	-
Step length (cm)	-	-	-	-	-
Right	32.81 ± 1.97	38.77 ± 2.75	30.67 ± 2.18	2.613	0.102
Left	37.45 ± 2.08	35.75 ± 5.35	36.97 ± 2.39	0.076	0.927
t	1.437	0.810	1.828	-	-
P	0.185	0.463	0.141	-	-
Stride length (cm)	-	-	-	-	-
Right	70.41 ± 2.50	75.29 ± 8.18	68.03 ± 3.26	0.561	0.581
Left	70.17 ± 2.80	74.32 ± 7.14	67.00 ± 2.78	0.611	0.554
t	0.198	0.745	0.585	-	-
P	0.847	0.498	0.590	-	-
Cadence (steps/mins)	-	-	-	-	-
Right	143.49 ± 5.06	139.87 ± 12.79	147.38 ± 7.19	0.181	0.836
Left	144.06 ± 5.98	139.71 ± 12.11	148.79 ± 7.13	0.245	0.786
t	0.356	0.217	0.652	-	-
P	0.730	0.839	0.550	-	-
Step interval (cm)	-	-	-	-	-
Right	15.81 ± 3.80	11.38 ± 2.09	15.08 ± 2.93	0.368	0.698
Left	15.21 ± 2.73	9.38 ± 1.23	15.97 ± 3.77	1.215	0.321
t	0.371	1.102	0.429	-	-
P	0.719	0.332	0.690	-	-

*Group A* left knee ROM similar to right knee ROM, *Group B* left knee ROM superior to right knee ROM, *Group C* left knee ROM inferior to right knee ROM, *y* years, *kg* kilogram. *cm* centimeter, *min* minute

The average right knee FL-EX distance (11.01 ± 0.53 cm, *n* = 20) was similar to that (10.94 ± 0.37 cm, *n* = 20) on the left (t = 0.173, *P* = 0.864). The mean hip FL-EX distance on the right side (2.98 ± 0.19 cm, *n* = 20) was comparable to that (2.73 ± 0.17 cm, *n* = 20) on the left (t = 1.172, *P* = 0.256). No significant correlation existed between the right and left knee and hip ROM in any group (*P* > 0.05) (Table 3).

**Table 3:** Correlation of right and left knee and hip ROM in each group

			Correlation Coefficient	*P*
Group A (*n* = 10)	Right knee ROM (cm)	11.30 ± 0.57	0.047	0.898
	Right hip ROM (cm)	3.43 ± 0.23		
Group A (*n* = 10)	Left knee ROM (cm)	11.09 ± 0.58	-0.063	0.864
	Left hip ROM (cm)	3.13 ± 0.19		
Group B (*n* = 5)	Right knee ROM (cm)	8.36 ± 0.42	-0.315	0.606
	Right hip ROM (cm)	2.04 ± 0.24		
Group B (*n* = 5)	Left knee ROM (cm)	10.72 ± 0.35	0.584	0.301
	Left hip ROM (cm)	2.56 ± 0.30		
Group C (*n* = 5)	Right knee ROM (cm)	13.07 ± 0.92	-0.167	0.788
	Right hip ROM (cm)	3.00 ± 0.29		
Group C (*n* = 5)	Left knee ROM (cm)	10.86 ± 0.94	0.471	0.423
	Left hip ROM (cm)	2.11 ± 0.36		

*ROM* Range of motion, *Group A* left knee ROM similar to right knee ROM, *Group B* left knee ROM superior to right knee ROM, *Group C* left knee ROM inferior to right knee ROM, *cm* centimeter

### MRI examination

The average acquisition time for R2* sequences was 29.15 ± 1.18 s (range, 25 to 42), which was significantly lower than that for T2 mapping sequences (504 s) (*P* < 0.001) (Table 1).

**Fig. 4:**
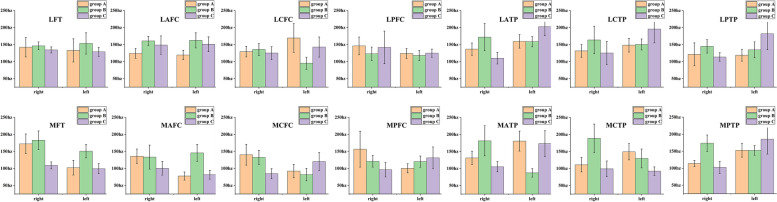
The R2* values (means and errors) of 28 cartilage ROIs in Groups A, B and C. LFT: lateral femoral trochlea; LAFC: lateral anterior femoral condyle; LCFC: lateral central femoral condyle; LPFC: lateral posterior femoral condyle; LATP: lateral anterior tibia plateau; LCTP: lateral central tibia plateau; LPTP: lateral posterior tibia plateau; MFT: medial femoral trochlea; MAFC: medial anterior femoral condyle; MCFC: medial central femoral condyle; MPFC: medial posterior femoral condyle; MATP: medial anterior tibia plateau; MCTP: medial central tibia plateau; MPTP: medial posterior tibia plateau

**Fig. 5:**
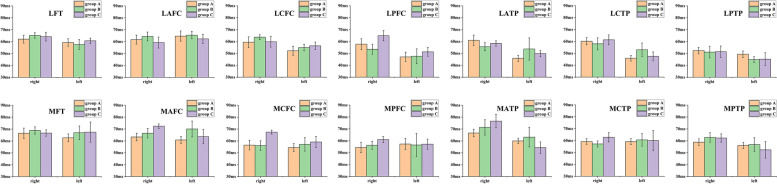
The T2 mapping values (means and errors) of 28 cartilage ROIs in Groups A, B and C. LFT: lateral femoral trochlea; LAFC: lateral anterior femoral condyle; LCFC: lateral central femoral condyle; LPFC: lateral posterior femoral condyle; LATP: lateral anterior tibia plateau; LCTP: lateral central tibia plateau; LPTP: lateral posterior tibia plateau; MFT: medial femoral trochlea; MAFC: medial anterior femoral condyle; MCFC: medial central femoral condyle; MPFC: medial posterior femoral condyle; MATP: medial anterior tibia plateau; MCTP: medial central tibia plateau; MPTP: medial posterior tibia plateau

A total of 1120 ROIs (28 per monkey) were evaluated in T2 mapping and R2* sequences. The MRI evaluations of each ROI showed excellent reliability for both T2 mapping and R2* sequences, with average ICCs of 0.90 ± 0.01 (range, 0.791 to 0.961) and 0.99 ± 0.00 (range, 0.978 to 0.997), respectively, in agreement with a previously published study [8]. One-way ANOVA indicated that significant differences in ROI values measured in the R2* sequence among the three groups existed for the cartilage of the right MPTP (*F* = 5.216, *P* = 0.017) and left MAFC (*F* = 4.919, *P* = 0.021) but not in the remaining 26 ROIs (*P* > 0.05) (Table 4). However, there were no significant differences in the 28 ROIs values measured with T2 mapping sequences among the three groups (*P* > 0.05). Among 560 ROIs in 20 monkeys, chi-square analysis indicated that the number (40 ROIs; 7.14%) of positive ROI R2* sequences reflected was significantly superior to that (0 ROIs) of positive T2 mapping sequences (χ^2^ = 41.481, *P* < 0.001); microstructural cartilage changes occurred more often in the medial knee compartment (40 ROIs; 14.29%) than in the lateral knee compartment (0 ROIs) (χ^2^ = 43.077, *P* < 0.001); and no significant difference was found between the right (20 ROIs; 7.14%) and left (20 ROIs; 7.14%) knee joints regarding the locations of microstructural cartilage changes (χ^2^ < 0.001; *P* = 1.000). Detailed data are shown in Figs. 4 and 5.

**Table 4:** Analysis of cartilage R2* values of the right MPTP and left MAFC among three Groups

	Group A (*n* = 10)	Group B (*n* = 5)	Group C (*n* = 5)	F	*P*
Cartilage R2* value of right MPTP (hz)	113.94 ± 9.01	173.47 ± 24.18	102.48 ± 17.67	5.216	0.017^#^
Cartilage R2* value of left MAFC (hz)	77.73 ± 11.91	145.74 ± 25.26	81.66 ± 13.04	4.919	0.021^#^

*MPTP* medial posterior tibia plateau, *MAFC* medial anterior femoral condyle, *Group A* left knee ROM similar to right knee ROM, *Group B* left knee ROM superior to right knee ROM, *Group C* left knee ROM inferior to right knee ROM, *hz* Hertz

#***:**** P* < 0.05

Pearson’s correlation analysis showed that the Group B cartilage R2* value of the right MPTP significantly increased with the absolute difference between right and left knee ROM (*r* = 0.913, *P* = 0.030). However, no significant correlation existed between the Group C cartilage R2* value of the left MAFC and ROM reduction (*r* = 0.199, *P* = 0.748).

## Discussion

In our study, we identified cartilage microstructural changes as a risk factor for reduced ROM through abnormal cartilage R2* values obtained in *cynomolgus monkeys* with limited knee kinematics. Our study revealed that MRI R2* sequences were superior to T2 mapping in detecting cartilage microstructural changes in knees with limited ROM, and the corresponding cartilage R2* value increased in proportion to the amount of knee ROM loss, which confirmed our previous hypothesis. However, other factors influencing ROM and progression of osteoarthritis such as synovial disease, joint effusions, and ligament stiffness could not be evaluated. In addition, although limitation of knee ROM is one of the effects of osteoarthritis, there is also evidence that osteoarthritis also progresses due to limited ROM [1, 2].

The present study showed that MRI-R2* sequences were a potential tool for the detection of microstructural cartilage changes in knees with limited ROM, with better superiority to T2 mapping. The superiority could be explained by the fact that the main microstructural changes in knee cartilage are related to iron overload around chondrocytes, to which R2* sequences are particularly sensitive [13, 18, 19]. Yao et al. [14] recently showed that chondrocyte ferroptosis, caused by an abnormal increase in iron deposition, can induce microstructural changes in cartilage. In our study, we found that the mean R2* values of cartilage ROIs of monkeys with limited knee FL-EX were significantly higher than those of monkeys with normal knee ROM. To date, no previously published studies have reported the MRI characteristics of microstructural cartilage changes in knees with limited ROM on R2* sequences [20]. In addition to the superiority, the acquisition time (< 50 s) needed for the R2* sequence was relatively less than that needed for the (> 500 s) T2 mapping sequence.

Our study also indicates that T2 mapping sequences exhibits some limits in the detection of microstructural cartilage changes in knees with limited ROM, which is different from previously published results [8]. This difference could be attributed to the imbalanced distribution of samples reported by Soellner et al. [8]. In their study, 92.5% of the patients had mild to moderate ROM loss, while only 1% had severe clinical symptoms. Several previous studies have illustrated that T2 mapping values are susceptible to magnetic disturbance, especially in patients with deeper microstructural cartilage changes [21, 22]. Shao et al. [21] reported that T2 mapping values for the middle to deep layers of cadaveric patellae were three times greater than those for the superficial layer. Link et al. [22] reported similar findings. In our study, the ages of all the samples were normally distributed, and the inferiority of T2 mapping may be attributed to many samples having deeper layer microstructural cartilage changes.

We also found that the medial knee compartment was more susceptible to microstructural cartilage changes than other regions, in agreement with the results of Sharma et al. [23]. In their study, the incidence (31%) of medial compartments with ROM loss in lower extremities with mild varus alignment was higher than that (22%) of lateral compartments with ROM loss in lower extremities with similar valgus alignment [23]. This is probably related to the fact that the medial knee compartment bears more load than the lateral compartment during activities of daily living. Several previous studies supported this hypothesis [24, 25]. Morrison [24] evaluated the mechanics of 14 normal knee joints and found that load was disproportionately transmitted to the medial compartment during walking. Nissan [25] reported similar results. In addition, previous studies have demonstrated that an increased load accelerates the progression of ROM loss [26, 27].

The current study indicated that the R2* values of cartilage ROIs increased in proportion to the amount of knee ROM loss. Our results partially agreed with previous studies [28–31]. Bittersohl et al. [28] reviewed 40 lateral femoral condylar cartilage specimens and found a significant decrease in T2* (1/R2*) values (< 100 ms) with increasing grade of cartilage degeneration (*P* < 0.001). Bittersohl et al. [29] found similar results. This finding could be related to the increased iron deposition and decreased water content of chondrocytes in patients with progressive ROM loss. However, Tsai et al. [30] and Marik et al. [31] reported conflicting results. In their study, T2* values increased with the severity of cartilage degeneration. These discrepancies could be attributed to inconsistent imaging sequences and parameters. In the studies reported by Tsai et al. [30] and Marik et al. [31], multiecho gradient-recalled echo (GRE) sequences where the time of repetition (TR) exceeded 400 ms were used; nevertheless, Bittersohl et al. [28, 29] utilized three-dimensional GRE-based sequences with TRs less than 100 ms. Similarly, our study used sequences with TRs less than 100 ms. Despite the inconsistent TRs, our study found that the cartilage R2* values could reflect the degree of microstructural cartilage changes.

Notably, our study measured knee ROM in a walking posture rather than a static posture. This was due to the increased load on the articular surface in a walking posture compared to a static posture. Morrison [24] reported that the maximum force on the knee articular surface during walking was 3 times the body weight measured in a static posture. Increased loading tends to be associated with greater knee ROM limitations, which would reflect the real pathological condition of microstructural cartilage changes.

There are some limitations in the present study. First, intraoperative and histological validation of T2 mapping and R2* sequences could not be performed. Second, it was not possible to evaluate other factors leading to limitation of knee joint kinematics, including synovial inflammation, effusion, and ligament stiffness. In addition, although limitation of knee ROM is one of the effects of osteoarthritis, there is also evidence that osteoarthritis also progresses due to limited ROM [1, 2].

Despite these limitations, microstructural cartilage changes were identified as a risk factor for reduced knee ROM due to the abnormal cartilage R2* values, and our study is the first to report the MRI characteristics of microstructural cartilage changes in R2* sequences and evaluate the difference between T2 mapping and R2* sequences in the detection of microstructural cartilage changes in monkeys with limited knee ROM. Third, only sagittal plane MRI sequences were used for analysis; however, previous studies used the same methodological approach [8, 30].

## Conclusions

In conclusion, R2* sequences show better strengths and shorter acquisition times than T2 mapping in the detection of microstructural cartilage changes in knees with limited ROM. The medial knee compartment was more susceptible to microstructural cartilage changes than other regions. The R2* value of knee cartilage tends to increase in proportion to the amount of ROM loss.

## Data Availability

The datasets used and analysed during the current study could be available from the corresponding author on request.

## Abbreviations

MRI: Magnetic Resonance Imaging
ROM: Range Of Motion
ROI: Region Of Interest
L: Lateral, M: Medial
A: Anterior
C: Central
P: Posterior
FT: Femoral Trochlea
FC: Femoral Condyle
TP: Tibial Plateau
LFT: Lateral Femoral Trochlea
LAFC: Lateral Anterior Femoral Condyle
LCFC: Lateral Central Femoral Condyle
LPFC: Lateral Posterior Femoral Condyle
LATP: Lateral Anterior Tibial Plateau
LCTP: Lateral Central Tibial Plateau
LPTP: Lateral Posterior Tibial Plateau
MFT: Medial Femoral Trochlea
MAFC: Medial Anterior Femoral Condyle
MCFC: Medial Central Femoral Condyle
MPFC: Medial Posterior Femoral Condyle
MATP: Medial Anterior Tibial Plateau
MCTP: Medial Central Tibial Plateau
MPTP: Medial Posterior Tibial Plateau
AAALAC: Association for Assessment and Accreditation of Laboratory Animal Care
m: Meter
km/h: Kilometer per hour
FL-EX: Flexion–Extension
cm: Centimeter
ICC: Intraclass Correlation Coefficient
GRE: Gradient-Recalled Echo
TR: Time Of Repetition
